# Autophagy in asthma and chronic obstructive pulmonary disease

**DOI:** 10.1042/CS20210900

**Published:** 2022-05-24

**Authors:** Peter J. Barnes, Jonathan Baker, Louise E. Donnelly

**Affiliations:** National Heart and Lung Institute, Imperial College London, U.K.

**Keywords:** aggresomes, inflammation, lysosome, macroautophagy, mitophagy, xenophagy

## Abstract

Autophagy (or macroautophagy) is a key cellular process that removes damaged molecules (particularly proteins) and subcellular organelles to maintain cellular homeostasis. There is growing evidence that abnormalities in autophagy may contribute to the pathogenesis of many chronic diseases, including asthma and chronic obstructive pulmonary disease (COPD). In asthma, increased autophagy plays a role in promoting type 2 immune responses and eosinophilic inflammation, whereas decreased autophagy may be important in neutrophilic asthma. Acute exposure to cigarette smoke may activate autophagy, resulting in ciliary dysfunction and death of airway epithelial cells, whereas in stable COPD most studies have demonstrated an impairment in autophagy, with reduced autophagic flux and accumulation of abnormal mitochondria (defective mitophagy) and linked to cellular senescence. Autophagy may be increased or decreased in different cell types and depending on the cellular environment, making it difficult to target autophagy therapeutically. Several existing drugs may activate autophagy, including rapamycin, metformin, carbamazepine, cardiac glycosides and statins, whereas others, such as chloroquine, inhibit this process. However, these drugs are nonspecific and more selective drugs are now in development, which may prove useful as novel agents to treat asthma and COPD in the future.

## Introduction

Autophagy (derived from the Greek auto: “self” and phagein: “to eat”) is a critically important cellular process that results in the removal of damaged molecules and subcellular organelles by lysosomes, to maintain cellular and protein homeostasis (proteostasis) and to allow recycling of their components. Autophagy plays and important role in development and cellular differentiation, but abnormalities in autophagy may contribute to disease and may be a target for new therapies (autophagy modulators) [1]. Autophagy plays an important role in the ageing process and cellular senescence and this process is impaired in many progressive age-related diseases [2]. Activation of autophagy prolongs lifespan in all species, from yeast to mammals, and is an important response to starvation. It is a highly regulated and evolutionary conserved process that engages lysosomes to degrade damaged organelles (such as mitochondria) and misfolded, aggregated or damaged proteins, in order to maintain the interior of the cell. There is a baseline nonselective autophagy process that engulfs cytoplasm to remove damaged proteins and organelles that keep the cytoplasm healthy and to recycle molecular components. This recycling process includes molecules, such as amino acids and nucleosides, from degraded proteins and DNA and as well as damaged organelles. Three main types of autophagy are recognised; *macroautophagy* (usually called autophagy), *microautophagy* and *chaperone-mediated autophagy* (CMA). Macroautophagy is the major mechanism of autophagy and involves the recycling of damaged proteins and organelles with transient double membrane vesicles called autophagosomes, which fuse with lysosomes, resulting in the degradation of its contents by lysosomal acid hydrolases. Microautophagy is a nonselective process that engulfs cytoplasmic elements in autophagic tubes before fusion and degradation by lysosomal enzymes. CMA is a selective form of autophagy, involving binding to the hsp-70 complex, that results in protein degradation by lysosomes to regenerate amino acids and plays an important role in cell metabolism. Disruption of autophagy pathways results in the accumulation of damaged proteins and organelles within the cytoplasm, giving rise to mitochondrial dysfunction, genomic instability and the generation of reactive oxygen species (ROS).

Autophagic pathways may have some specificity. *Mitophagy* refers to the selective degradation and removal of damaged mitochondria in order to maintain normal mitochondrial function [3]. Other damaged organelles may also be selectively removed by autophagy, including endoplasmic reticulum (*ER-phagy*), nucleus (*nucleophagy*) and lysosomes themselves (*lysophagy*). *Lipophagy* involves the uptake and degradation for lipid droplets, whereas *aggregaphy* is the removal of protein aggregates. *Xenophagy* is an autophagic process directed against intracellular pathogens, such as viruses and bacteria. Autophagy is mainly involved in removal of defective intracellular organelles and the removal of long-lived proteins or protein aggregates, whereas the 26S ubiquitin-proteasome system is involved in turnover of more short-lived proteins. Autophagy, by keeping the cell healthy, plays an important role in repair and allows survival under adverse conditions, such as nutrient depletion, whereas inhibition of autophagy may lead to prolonged cell survival (cellular senescence) or to cell death. Inhibition of autophagy results in inflammation through the activation of the NLRP3 inflammasome [4]. Abnormal autophagy plays a critical role in the pathogenesis of many diseases, including cancer, neurodegenerative diseases, atherosclerosis and chronic inflammatory diseases [5]. There is increasing evidence that autophagy also plays an important role in several chronic lung diseases and may be a target for new therapies [6]. Here we review the role of macroautophagy in asthma and chronic constructive pulmonary disease (COPD), which are the most prevalent lung diseases.

## Molecular mechanisms of autophagy

**Figure 1:**
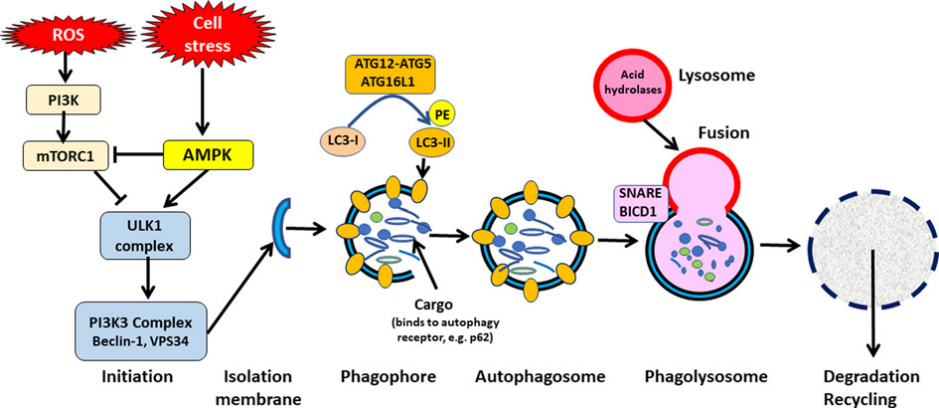
Autophagy pathways Autophagy is initiated by the ULK-1 kinase complex which is activated by AMPK and inhibition of mTORC1. The ULK-1 complex activates a PI3K class III complex, which includes Beclin-1 and VPS34, which results in the development of an isolation membrane, which elongates and recruits the ATG5-ATG12-ATG16-L1 complex, which converts LC3-I to LC3-II through the interaction with phosphatidylethanolamine (PE). LC3-II binds to autophagy receptors, such as p62, which are bound to cargo proteins and organelles designated for degradation. Autophagosomes then fuse with lysosomes via SNARE proteins and BICD1 to form autolysosomes. The cargo is then degraded by lysosomal acid hydrolases and the degradation products (such as amino acids) are recycled.

The molecular pathways that induce autophagy involve several autophagy related (Atg) proteins were first were identified in yeast and are evolutionarily conserved [7]. The formation of the double membrane autophagosome vesicle is complex and involves 16 Atg proteins and two ubiquitin-like conjugation systems that control several autophagy regulators, resulting in the fusion of autophagosomes with lysosomes and the degradation of their content (Figure 1). Autophagy is initiated by inhibition of the mechanistic target of rapamycin complex 1 (mTORC1), which activates unc-51 like activating kinase 1 (ULK1), which translocates to the endoplasmic reticulum and forms a complex with various Atg proteins. It also activates a Class III phosphoinositide-3-kinase (PI3K) complex that includes Beclin-1 (Atg6) and this results in formation of a phagophore, which is a crescent shaped double membrane within the cytoplasm. The PI3K complex which contains VPS-34 kinase (vacuolar protein sorting 34, encoded by PIK3C3) phosphorylates and induces the nuclear localization of the transcription factor EB (TFEB), which switches on several autophagy and lysosome genes. Microtubule-associated light chain protein-3 (LC3-I) is a ubiquitin-like protein that is involved in elongation and closure of the phagophore to form the autophagosome vesicle through conjugation with phosphatidylethanolamine (PE), which converts LC3-I to LC3-II. Another complex comprising Atg-12, Atg-5 and Atg-16LI has E3 ubiquitin activity against the LC3-PE complex that results in closure of the autophagosome. Autophagosomes express several proteins, including SNAP29, which interact with molecules expressed on lysosomes, resulting in fusion to form an autolysosome and subsequent degradation of the autophagosome cargo by lysosomal acid hydrolases. The contents of the lysosome, including nutrients, such as amino acids, and LC3 are then recycled.

### Activating mechanisms

Autophagy may be activated by different cellular stimuli, such as nutrient deficiency, pathogens, toxins and oxidative stress, which activate AMP kinase (AMPK), which in turn activates ULK-1 to initiate autophagy. AMPK is also a key inhibitor of mTORC1, which normally inhibits autophagy. Calmodulin-dependent protein kinase-2 may also initiate autophagy via ubiquitination of Beclin-1 [8]. The antiaging molecule sirtuin-1 (SIRT1) activates FOXO transcription factors, which may also activate autophagy and are be reduced by mTORC1 activation. p62 (SQSTM1, sequestrome-1) is a selective autophagy receptor, which functions as an adaptor protein that binds ubiquitinated proteins that are designated for degradation by the 26S proteasome, and also binds LC3, resulting in autophagosome formation. The cargo is thereby engulfed within the autophagosome vesicle so that it can be transported to the lysosome [9]. Other selective autophagy receptors include NBRI (neighbour of BRACA gene-1), optineurin and nuclear dot protein-52 (NDP52) have selectivity for different cargoes and are regulated by post-translational modifications by kinases and acetylases [10,11].

### Mitophagy

**Figure 2:**
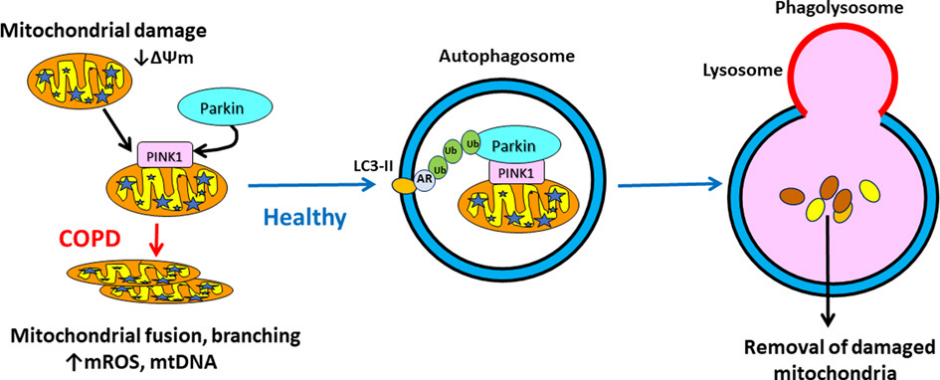
Mitophagy Damaged mitochondria are recognised by PTEN-induced putative kinase-1 (PINK1), which recruits Parkin, leading to ubiquitination (Ub) and engulfment into an autophagosome through binding to autophagy receptors (AR), such as p62, which binds to LC3-II. Fusion with a lysosome leads to degradation and removal of defective mitochondria in healthy cells. In COPD, Parkin is reduced so defective mitochondria accumulate in cells and may fuse and branch and release mitochondrial reactive oxygen species (mROS) and mitochondrial (mt)DNA.

Autophagy plays a key role in maintaining normal mitochondrial function by detecting mitochondrial damage and removing there damaged organelles [12]. The best characterized mitophagy mechanism involves the serine/threonine kinase PINK1 (PTEN-induced putative kinase)-Parkin signaling pathway. To maintain mitochondrial health PINK1 is transported to the inner mitochondrial membrane, where it is cleaved, but when mitochondria are depolarized or damaged, PINK-1 accumulates on the outer mitochondrial membrane and is autophosphorylated (Figure 2). This triggers the recruitment of Parkin, an E3 ligase that induces removal of mitochondria by autophagy. Mitophagy may also be induced independently of PINK1-Parkin signalling by other mitophagy receptors and E3 ligases. Cardiolipin is a lipid that is exclusively bound to the inner mitochondrial membrane and plays an important role in maintaining normal mitochondrial function and in the regulation of mitophagy. Mitochondrial dysfunction and impaired mitophagy are commonly found, particularly in airway epithelial cells, in COPD and asthma [13].

### Measuring autophagy

Autophagy can be measured by several complementary approaches. Electron microscopy can be used to demonstrate autophagosome vesicles, and LC3 punctae can be demonstrated by confocal microscopy [14]. Western blotting may be used to quantify autophagic proteins, such as LC3, LAMP-1 and Atgs. Autophagic flux is measured by the conversion of LC3-1 to LC3-II and p62 degradation. Autophagy may be inhibited by bafilomycin A1, chloroquine or hydroxychloroquine and induced by rapamycin and cell starvation [15].

## Autophagy in asthma

Asthma is characterised by eosinophilic inflammation of the airway mucosa that is orchestrated by T helper 2 (Th2) and type 2 (T2) innate lymphoid cells (ILC2) through the section of T2 cytokines, including interleukin(IL)-4, IL-5 and IL-13. This eosinophilic inflammation results in airway hyperresponsiveness (AHR) and variable airflow obstruction. Mast cells are recruited to the airway surface and release potent spasmogens, including histamine, cysteinyl-leukotrienes and prostaglandin D_2_ [16]. Autophagy plays an important role in the pathogenesis of atopy and asthma and may be detrimental or beneficial, depending on the cell types involved [17].

### Studies in experimental asthma

The T2 cytokine IL-13 induces goblet cell hyperplasia and mucus hypersecretion in mice and this is blocked in *Atg5*-deficient animals [18]. Ovalbumin (OVA) challenge in sensitised mice results in eosinophilic lung inflammation with increased LC3 expression and autophagosome formation in eosinophils. Inhibition of autophagy by *Atg5* knockdown with siRNA or treatment with the autophagy inhibitor 3-methyladenine (3-MA), which is a class III PI3K inhibitor, attenuates this allergic response [19]. Similarly, treatment with 3-MA prior to OVA challenge reduces airway inflammation, goblet cell hyperplasia and eosinophil extracellular trap formation [20]. In sensitised mice, inhibition of mTORC1 by rapamycin or gene knockout activates autophagy and this enhances allergic inflammation and increases epithelial cell expression of the alarmin IL-25, whereas *LC3B* knockout attenuates inflammation [21]. *Atg5* knockout in mice also reduces secretion of T2 cytokines from ILC2 cells [22]. Attenuation of autophagy by chloroquine administration in house dust mite-sensitized and exposed mice results in decreased allergic inflammation, AHR and airway remodelling, in association with a decrease in the expression of the *Atg5* and *Beclin-1* [23]. B-lymphocytes from ovalbumin-sensitized and exposed mice show increased autophagy that is mediated by IL-4 and enhanced antigen presentation [24].

These studies suggest that increased autophagy is associated with increased T2 inflammation in murine models of asthma. However, other studies suggest that autophagy may be impaired in some models. Conditional knockout of *Atg5* in mice suppresses autophagy and results in greater AHR and neutrophilic inflammation after exposure to house dust mite in sensitised animals [25]. This is associated with increased IL-17 expression and resistance to the anti-inflammatory effects of corticosteroids, suggesting that impaired autophagy may be important in non-T2 severe asthma. Further analysis suggests that impairment of autophagy in dendritic cells is critical to the development of this neutrophilic asthma model. In mice sensitized to ovalbumin by the transfer of sensitized IL-17 expressing Th17 cells there is induction of neutrophilic lung inflammation, which is suppressed by the autophagy activator rapamycin [26]. This supports the link between suppression of autophagy and neutrophilic inflammation after allergen exposure. *Atg7* deficiency in murine bronchiolar epithelial cells induces p62 accumulation and AHR to methacholine but no increase in inflammatory cells [27]. These discrepant results in mice showing beneficial and harmful effects of autophagy may be dependent on which cell types are involved but also by the kinetics of the inflammatory process, although mice may not be an appropriate animal model for human asthma.

### Studies in human asthma

In candidate gene association studies, polymorphisms of *Atg5* have been associated with asthma, including childhood asthma [28,29]. Polymorphisms of *Atg5* and *Atg7* are not linked to asthma susceptibility or severity, but are associated with neutrophilic inflammation in sputum, suggesting a link to non-T2 asthma [30]. Increased expression of ATG5 protein is found in airway epithelial cells in severe asthma and associated with subepithelial fibrosis and increased expression of collagen-1 [31]. Dysregulation of autophagy is linked to increased fibrosis in several chronic diseases, including idiopathic pulmonary fibrosis, cirrhosis and chronic kidney disease and may be linked to increased release of transforming growth factor-β (TGF-β). Activated fibroblasts from various tissues express *Atg5*, whereas knockdown of *Atg5* with siRNA protects against fibrosis [32]. IL-13 plays a key role in T2 asthma and blocking its receptor IL-4Rα with the antibody dupilumab is effective in controlling severe T2 asthma and preventing exacerbations [33]. IL-13 stimulates goblet cell formation and MUC5AC secretion from human airway epithelial cells *in vitro*, which is correlated with the induction of autophagy, with an increase in LC3-II and increased autophagic flux that is prevented by *Atg5* knockdown [18]. Blocking autophagy in these cells also inhibits the generation of reactive oxygen species (ROS) in response to IL-13, an effect mediated through the activation of the NADPH oxidase DUOX1 [34]. Exposure of human bronchial epithelial cells to particulates increases autophagy, with the accumulation of autophagosomes, accompanied by secretion of CXCL8 and increased expression of MUC5AC, which are prevented by knockdown of *Beclin-1* and *LC3B* [35]. Chronic exposure to *Alternaria* extracts also induces autophagy in human airway epithelial cells, with increased autophagosome formation, conversion of LC3-II and decreased p62. This is associated with increased secretion of IL-18, which is prevented by autophagy inhibitors 3-MA and bafilomycin-1A [36]. Exposure of airway epithelial cells from asthmatic patients to IL-13 or IL-33 activates autophagy through the inhibition of mTORC1 and is inhibited by *LC3B* knockdown [21].

There is an increase in autophagy (measured by increased LC3-II) in sputum and peripheral blood eosinophils and in blood neutrophils from patients with severe asthma, compared with non-severe and non-asthmatic individuals. In an eosinophil cell line (HL-60) IL-5 induces an increase in LC3-II and release of eosinophil cationic protein, which is inhibited by 3-MA [37,38]. IL-17 induces autophagy and mitophagy in bronchial fibroblasts from patients with severe but not non-severe asthma and is associated with a profibrotic phenotype, which was reversed by bafilomycin-A1 [39]. Bronchial fibroblasts from patients with severe asthma show increased mitophagy and expression of PINK-1 and Parkin, as well as an increased LC3-II expression and a profibrotic phenotype, perhaps as a compensatory response to mitochondrial dysfunction in asthmatic cells [40].

### Effects of therapy

Overall, autophagy is increased in key effector cells in asthma and this is linked to increased T2 inflammation and suppression of the anti-inflammatory cytokine IL-10. IL-10 inhibits starvation-induced activation of autophagy in murine macrophages via increased PI3K signaling and mTORC1 activation [41], whereas other studies have shown that IL-10 inhibits mTORC1 and thus induces autophagy in macrophages [42]. In monocyte-derived macrophages (MDM) cultured in macrophage colony-stimulating factor (M-CSF) and IL-4 to induce an M2-like phenotype, IL-10 markedly suppresses rapamycin-induced autophagy and autophagic flux to a similar extent to 3-MA [43]. IL-10 secretion by macrophages is reduced in patients with asthma, and this is reflected by a reduced concentration of IL-10 in induced sputum [44–46]. Corticosteroids increase the expression of IL-10 in macrophages, and this may contribute to their anti-inflammatory effects in asthma [47,48]. Asthmatic patients treated with inhaled corticosteroids show a reduction in macrophage autophagy (reduced sputum macrophage Beclin-1 and LC3) and autophagic flux (p62); this is correlated with increased sputum IL-10 and reduced IL-4 concentrations. This inhibitory effect of budesonide is confirmed *in vitro* in MDMs with an increase in IL-10 secretion, which further inhibits autophagy [43]. Suppression of autophagy by knocking down LC3 results in a marked increase in IL-10 expression and secretion. Statins may enhance the anti-inflammatory effects of corticosteroids in asthmatic patients though an increase in macrophage IL-10 expression [49]. Simvastatin potentiates the anti-inflammatory effects of budesonide by enhancing the inhibitory effects of corticosteroids on autophagy, with a greater increase in IL-10 and a reduction in IL-4 and sputum eosinophils. Inhibition of IL-10 with a blocking antibody or using siRNA to reduce its expression in macrophages reverses the inhibitory effect of budesonide and simvastatin [43]. Corticosteroids may inhibit autophagy through inhibition of TANK-binding kinase-1, which promotes autophagy through induction of autophagy proteins including p62 to reduce maturation of autophagosomes [50].

## Autophagy in COPD

COPD, now the fourth ranked cause of death globally and a common cause of acute hospital admission with exacerbations is associated with progressive airflow limitation and chronic inflammation of the lungs [51]. There is increasing evidence that accelerated lung ageing is an important driving mechanisms with the accumulation in the lung of senescent cells, enhanced by a loss of endogenous antiaging molecules, such as SRT1, due to increased oxidative stress in the lungs [52]. As discussed above, defective autophagy is commonly associated with accelerated ageing and cellular senescence, so may pay an important role in the pathogenesis of COPD. As in asthma, some studies have shown increased and other decreased autophagy in COPD and experimental models which may reflect different cell types and environmental conditions [58]. One study has shown that one polymorphism of an autophagy gene (*Atg16L1*) is associated with more than 3-fold increased risk of developing COPD [53].

### Experimental studies

Mice exposed chronically to cigarette smoke develop emphysema and increased expression of Toll-like receptor (TLR4), but *Tlr4* deficiency further enhances the development of emphysema and is associated with increased expression of autophagy markers, such as LC3 [54]. Particulate aerosols induce lung inflammation in mice that is enhanced by *Mtor* deficiency, and reduced when autophagy is inhibited by *Atg5*-deficiency, suggesting that increased autophagy contributes to lung inflammation in this model [55]. Cigarette smoke exposure impairs mucociliary function and results in shortened and dysfunctional cilia in the airways of mice *in vivo* and in tracheobronchial cells *in vitro*, with increased autophagic turnover of ciliary proteins mediated by histone deacetylase (HDAC)6 [56]. Deficiency of *Beclin-1* prevents the reduction in mucociliary clearance after cigarette smoke, confirming the role of autophagy in this process. Cigarette smoke also induces mitochondrial dysfunction in airway epithelial cells, and stimulation of mitophagy, which results in cell death by necrosis (necroptosis) [57]. This is prevented by genetic deletion of PINK1, which protects mice from developing mitochondrial dysfunction and emphysema after exposure to cigarette smoke [58].

Most studies, however, indicate that autophagy mechanisms are impaired in COPD. Human bronchial epithelial and A549 cells acutely exposed to cigarette smoke *in vitro* show accumulation of polyubiquitinated proteins, indicating impaired proteostasis, and this is associated with increased ROS generation and cellular necrosis [59]. *In vitro* exposure of the bronchial epithelial cell line BEAS-2B to cigarette smoke extract induces ubiquitinated protein aggregates, which colocalize with LC3B and p62, which are reduced by the autophagy-inducing drug carbamazepine [60]. This is replicated in mice exposed to cigarette smoke, with increase in aggresomes, LC3B and p62 in peripheral lung tissue, which correlates with increased cellular senescence [61]. In mice exposed to cigarette smoke the increase in pulmonary p62 is strongly correlated with increased expression of bicaudal D1 (BICD1), an adaptor protein that plays a key role in binding to the dynein motor machinery linking transport of vesicles by microtubules to lysosomes [62]. This is inhibited by carbamazepine treatment, which reduces the development of emphysema in mice [60]. A single-nucleotide polymorphism in the dynein-binding region of BICD1 has been described as a risk factor for emphysema [63]. Deficiency of Parkin, a critical regulator of mitophagy, in mice results in impaired mitochondrial function, with increased airway wall thickening and emphysema after cigarette smoke exposure, indicating that defective mitophagy may contribute to the development of experimental COPD [64].

### Studies in COPD patients

Peripheral lung tissue from patients with severe COPD show an increase in p62, LC3 and aggresomes compared with age-matched non-smokers, suggesting an impairment of autophagy in COPD [60,61,65]. The increase in p62 in peripheral lung of COPD patients is related to disease severity and is strongly correlated with increased expression of LC3 and BICD1 [62]. Similarly, alveolar macrophages from COPD patients and smokers show an increase in p62, increased numbers of autophagosomes and mitochondrial dysfunction, with impaired autophagic flux in COPD [62,65,66]. This is mimicked by exposure of alveolar macrophages to cigarette smoke extract *in vitro*, with accumulation of LC3, ubiquitinated proteins and aggregates, and with reduced autophagic flux. These cells also show reduced uptake of labelled bacteria (*Escherichia coli*) and reduced delivery of bacteria to lysosomes, indicating a defect in xenophagy [66]. Cigarette smoke extract activates autophagy in a human macrophage cell line with an increase in autophagosomes but impairs autophagic flux (measured by LC3 turnover and bafilomycin-A1, resulting in accumulation of the autophagy receptor NDP52, which interacts with galectin-8 [67]. This is associated with increased expression and secretion of galectin-8, a danger signal that which identifies damaged intracellular vesicles to initiate autophagy [68]. Galectin-8 is increased in lungs and plasma of COPD patients [67]. Human bronchial epithelial cells exposed to cigarette smoke extract *in vitro* show an initial increase in autophagy, followed by impairment and accumulation of p62 and ubiquitinated proteins and induction of cellular senescence [65]. Autophagy inhibition with 3MA and LC3 or *Atg5* knockdown results in p62 accumulation and further cellular senescence. Cigarette smoke extract induces autophagy in BEAS-2B and primary human bronchial epithelial cells with increased expression of p62 oligomers, LC3 and autophagosomes, with evidence for a defect in autophagosome maturation, with a failure of autophagosomes to fuse with lysosomes [62]. Overexpression of BICD1 inhibits autophagosome maturation, whereas its knockdown decreased accumulation of p62 and LC3 [62]. Autophagic flux may also be impaired because of dysfunctional lysosomes, with evidence for increased leakiness of lysosomes and failure of their removal by lysophagy, which is mediated by an interaction between galectin-3 and tripartite motif protein (TRM)-16 [69]. Cigarette smoke extract impairs lysophagy in human primary epithelial cells *in vitro*. Airway epithelial cells from COPD patients show increased aggresomes, which stain for galectin-3 punctae, whereas TRM-16 is reduced, indicating the accumulation of defective lysosomes, linked to increased cellular senescence [70].

Mitochondrial dysfunction is commonly observed in pulmonary cells from COPD patients, with increased numbers of abnormal mitochondria that show reduced mitochondrial membrane potential, increased release of mitochondrial ROS and decreased ATP formation [71,72]. Parkin expression is reduced in COPD lung tissue and knockdown of PINK1 and Parkin in BEAS-2B and primary bronchial epithelial cells results in reduced mitophagy, increased mitochondrial ROS production and cellular senescence, indicating that impaired mitophagy may be important in the pathogenesis of COPD [73]. Conversely, overexpression of Parkin in BEAS-2B cells abrogates mitochondrial dysfunction and cellular senescence induced by cigarette smoke extract *in vitro* [73].

Impairment of autophagy may also reduce the clearance of intracellular bacteria through xenophagy in alveolar macrophages of smokers [66] and combined with a defect in bacterial phagocytosis in macrophages from COPD patients this may account for bacterial colonization of the lungs in COPD patients and for an increased susceptibility to exacerbations [74,75]. There may be a link between defective mitophagy and impaired bacterial phagocytosis as this is linked to impaired mitochondrial function in COPD macrophages [76].

## Therapeutic implications

**Figure 3:**
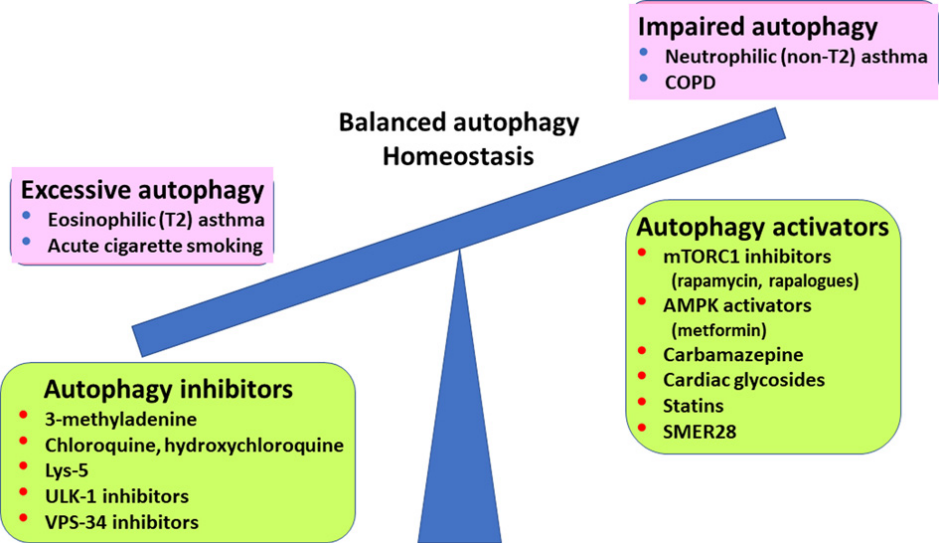
Imbalance in autophagy and potential autophagy modulators Increased autophagy may be seen in type 2 (T2) asthma and with acute exposure to cigarette smoke and may be reduced by autophagy inhibitors, which restore autophagic flux to normal. Decreased autophagy is seen in COPD and non-T2 asthma and may be restored by various autophagy activators.

There is growing evidence that abnormal autophagy contributes to the pathophysiology of asthma and COPD, suggesting that restoring autophagy to normal to maintain cellular homeostasis would be of therapeutic benefit. Several drugs have been shown to increase or decrease autophagy, although these drugs lack specificity so have other actions that may contribute to their beneficial and adverse effects. No drugs have been developed that specifically target autophagy. A major problem is that autophagy may affect different cell types in different ways so that the overall effect of a drug may be unpredictable. To some extent this can be addressed using cell-specific deletion of autophagy-related genes and several such mouse models are already available [77]. Also, both increases and decreases in autophagy have been described in asthma and COPD. This may be related to the severity and duration of a particular cellular stress, the cell types involved and different pheno-endotypes of asthma and COPD. For example, autophagy appears to be increased in eosinophilic inflammation in asthma, so that inhibitors should be beneficial, whereas it is impaired in severe neutrophilic asthma, where activators may be indicated. In COPD acute stress with cigarette smoke may increase autophagy, whereas in chronic disease autophagy is usually impaired (Figure 3).

Several classes of drug have been found to increase or decrease autophagy and act at different points in the autophagic pathway so that combination therapies are possible [78]. In addition, dietary interventions and lifestyle changes may also have beneficial effects on autophagy and represent a complementary approach. The effects of drug interventions may be difficult to predict and interpret as most of the drugs used have other effects on the cell. For example, rapamycin, which activates autophagy, may also reduce cellular senescence, which is closely linked to autophagy [2].

### Autophagy activators

Several drugs have been shown to increase autophagy when it is reduced in disease and so may be indicated in the treatment of stable COPD and some patients with severe non-T2 asthma. Inhibition of mTORC1 by rapamycin and related rapalogues (including temsirolimus, everolimus and umerolimus) activates autophagy. mTOR is activated in COPD lungs and overexpression of mTORC1 in alveolar epithelial cells results in the rapid development of emphysema in mice [79,80]. Rapamycin inhibits the increased mTOR activation in COPD lung endothelial cells and reduces the expression of proinflammatory mediators, such as IL-6 and CXCL8. Orally administered rapamycin protects against the development of emphysema after cigarette smoke exposure in mice with overexpression of mTORC1 [80]. These effects of rapamycin are associated with reduced cellular senescence and improved mitochondrial function and are presumably mediated through an increase in autophagy. By contrast, another study showed that mTOR was reduced in airway epithelial cells from COPD patients and after exposure to cigarette smoke extract *in vitro*. In mice with selective knockdown of *mTOR* in epithelial cells, cigarette smoke exposure increased inflammation and airspace enlargement [81]. By contrast, in mice sensitized and exposed to ovalbumin rapamycin reduced neutrophilic inflammation due to an inhibitory effect on Th17 cells, consistent with an impairment in autophagy in neutrophilic asthma [82]. Rapamycin is also effective in a conventional model of allergic asthma in mice, with a reduction in lung eosinophils and a reduction in eosinophil differentiation [83]. However, there are no studies that have specifically measured the effects of rapamycin on autophagy in COPD or asthma, and there are no reports of the effects of mTORC1 inhibitors in clinical studies in these diseases. Although rapamycin and rapalogues are now used to treat the rare lung disease lymphangioleiomyomatosis, these treatments have significant adverse effects, but lower doses than required for immunosuppression might be effective in inducing autophagy and cellular senescence [84].

Autophagy may also be activated by AMPK activators, which also inhibit mTORC1. Although several AMPK activators have been developed [85], only metformin has been tested in clinical studies as it is widely used as to treat type 2 diabetes. Metformin reduces exacerbations in patients with asthma [86] and reduces inflammation and structural changes in response to allergen challenge in sensitized mice through activation of AMPK [87]. Metformin also protects mice against cigarette smoke-induced emphysema and airway structural changes and was associated with a reduced progression of COPD in an epidemiological study [88]. However, these studies have not yet linked the beneficial effects of metformin specifically to activation of autophagy and may be explained by other effects of this drug.

Carbamazepine is an anticonvulsant that activates autophagy through inhibiting PI3K signaling [78]. In mice exposed to cigarette smoke carbamazepine inhibits the accumulation of aggresomes in the lung and prevents the development of emphysema [60]. No clinical trials of carbamazepine in COPD have been reported. Cardiac glycosides, such as digoxin, which inhibit Na^+^-K^+^-ATPase, also improve defective autophagy in airway epithelial cells that show impairment in autophagosome maturation after cigarette exposure though reducing BICD1 and p62 accumulation [62]. SMER28 is a small molecule that induces autophagy independently from mTORC1 also increases autophagy in these cells [62].

Statins, such as atorvastatin, have been shown to induce and inhibit excess autophagy, depending on the cell type and cellular stimulus [89]. In ovalbumin-sensitized and exposed mice, simvastatin increases the expression of autophagy proteins ATG5, LC3B and Beclin-1 and the number of autophagosomes in lung tissue, with concomitant suppression of IL-4, IL-5 and IL-13, and reduction in extracellular matrix [90]. These effects of the statin are reversed by 3-MA. Statins may have a beneficial effect in patients with asthma, with improvement in asthma control [91]. A statin has a beneficial effect in smoking asthmatic patients who usually have non-T2 asthma [92], although it cannot be assumed that this benefit is mediated through increased autophagy. However, as discussed above a statin may enhance the anti-inflammatory effects of an inhaled corticosteroid in asthmatic patients through an effect on autophagy [43]. Similarly, statins may also benefit patients with COPD and decrease neutrophilic inflammation [93].

Since oxidative stress appears to be an important mechanism that impairs autophagy in COPD and asthma, antioxidants may be an indirect means of restoring normal autophagic flux. However, current antioxidants, such as *N*-acetylcysteine, are poorly effective in COPD and there is a search for more effective and better tolerated antioxidants [52].

### Autophagy inhibitors

Where increased autophagy contributes to airway disease pathogenesis autophagy inhibitors may be beneficial. This may therefore be an approach to inhibiting eosinophilic inflammation in asthma, which is potentiated by increased autophagy. In alveolar macrophages from asthma patients inhibition of autophagy by 3-MA induces the secretion of the anti-inflammatory cytokine IL-10 [43]. 3-MA inhibits autophagy via inhibition of Class III PI3Ks, thereby inhibiting the formation of autophagosomes, but is not suitable for clinical administration because of toxicity issues, such as induction of apoptosis [94]. Inhibition of autophagy may also be potentially beneficial in COPD by inhibiting the increase in autophagy that has been demonstrated in animal models and in airway epithelial cells after acute cigarette smoke exposure, so may be indicated in acute exacerbations [95]. Since other evidence suggests that autophagy is impaired in COPD it is not clear whether activation or inhibition of autophagy should be achieved. It is likely that autophagy pathways are differently affected in different cell types and also in response to different types of cellular stress, such as acute exposure to cigarette smoke or chronic low level exposure.

Chloroquine and hydroxychloroquine are well-tolerated drugs that inhibit autophagy by blocking lysosomal function but have numerous other pharmacological activities that makes them very nonspecific. A more specific lysosomal inhibitor Lys5 has been developed but, no studies in airway diseases reported. More specific autophagy inhibitors are in development, including the ULK-1 inhibitors SBI-0206965 and MRT68921, which are in development as cancer therapies [78].

## Conclusions and future developments

There remains uncertainty about the role of autophagy in the pathogenesis of asthma and COPD, which may reflect different responses in different cell types and the effects of different stimuli used for *in vitro* studies, as well as differences in experimental *in vivo* models. There may also be differences in the role of autophagy in different pheno-endotypes of asthma and COPD. For example, increased autophagy is associated with T2 inflammation in asthma, whereas neutrophilic asthma is associated with impaired autophagic flux (Figure 3). In COPD acute exposure to cigarette smoke increases autophagy and may lead to cell death, whereas chronic exposure is more likely associated with impaired autophagy, with impaired cellular function and increased cellular senescence. This raises an important issue in the development of modulators of autophagy in the potential treatment of asthma and COPD. Autophagy is a complex mechanism involving many components and is important for the maintenance of a healthy cellular environment, so the aims of therapeutic intervention are to achieve a balanced autophagy. There are currently no drugs that are specific for autophagy; each component has other cellular functions, so that its modulation may have several other consequences. For example, inhibition of mTORC1 not only activates autophagy but also inhibits the development of cellular senescence, improves mitochondrial function and reduces protein synthesis and cell proliferation [96]. This might be addressed in the future by combining drugs that target different steps in the autophagic processing. For example, combining drugs that activate autophagy upstream, such as AMPK activators or mTORC1 inhibitors with drugs that accelerate lysosomal degradation may be more effective in some types of autophagy defect.

Several existing drugs have been found to modulate autophagy, including rapamycin and rapalogues, metformin and chloroquine, but these drugs all have several other actions so are not specific for autophagy [97]. Cardiac glycosides, widely used in the treatment of cardiovascular diseases, are activators of autophagy, although under some conditions they may be inhibitory [98]. Because of the key role of autophagy in neurodegenerative disease, cardiovascular diseases and cancer, there is a concerted effort to discover more selective drugs [5]. More specific autophagy modulators, such as ULK-1 inhibitors [99] and selective inhibitors of VPS34, a Class III PI3K, are also effective inhibiters of autophagy and in development as cancer therapy [100]. Several naturally occurring compounds have also been found to have autophagy modulating effects, including traditional Chinese herbal medicines used for asthma treatment [101]. Defective mitophagy may be targeted by autophagy activators, such as metformin and rapamycin, but also more specifically by drugs that activate the PINK1/Parkin pathway [12]. A recent study suggests that cannabidiol activates PINK1/Parkin signaling and increases mitophagy [102]. Elamipretide is a mitochondrially targeted tetrapeptide that stabilises cardiolipin and maintains mitochondrial function, which is currently being studied in mitochondrial diseases and cardiac failure [103].

### Biomarkers

One problem in studying autophagy in human disease is the quantification of autophagy. Measuring single biomarkers, such as LC3 in cells or tissue may be misleading and it is more informative to measure autophagic flux. One approach is to measure lipidated LC3 linked to green fluorescent protein (GFP), so that fluorescent punctae can be quantified in the absence and presence of a lysosomal inhibitor such as bafilomycin-A1 [15]. This approach has been used to measure autophagy in peripheral blood mononuclear cells, so could be developed to measure autophagic flux in bronchoalveolar lavage or sputum cells for airway disease patients. A single tandem probe consisting of GFP- LC3- linked to red fluorescent protein (RFP)-LC3ΔG, is cleaved by ATG4 proteases and LC3-GFP is degraded by autophagy, whereas RFP-LC3ΔG persists in the cytoplasm as an internal control, so that the ratio of green to red fluorescence can be used to measure autophagic flux [104]. This assay could also be used for drug screening.

## Data Availability

Data sharing not applicable to this review.

## Open Access

Open access for this article was enabled by the participation of Imperial College London in an all-inclusive *Read & Publish* agreement with Portland Press and the Biochemical Society under a transformative agreement with JISC.

## Abbreviations

3-MA: 3-methyladenine
AHR: airway hyperresponsiveness
AMPK: AMP-activated protein kinase
ATG: autophagy-related genes
BAL: bronchoalveolar lavage fluid
BCL2: B-cell CLL/lymphoma 2
BICD1: bicaudal D1
CMA: chaperone-mediated autophagy
COPD: chronic obstructive pulmonary disease
CXCL: cysteine-X-cysteine chemokine ligand
DUOX: dual oxidase
FOXO: forkhead box O transcription factor
HDAC: histone deacetylase
hsp: heat shock protein
IL: interleukin
ILC2: type 2 innate lymphoid cell
LAMP: lysosomal-associated membrane protein
LC3: microtubule-associated protein 1 light chain 3
MDM: monocyte-derived macrophages
mTORC: mammalian target of rapamycin complex
NADPH: reduced nicotinamide adenine dinucleotide phosphate
NBRI: neighbor of BRACA gene-1
NDP: nuclear dot protein
NF-κB: nuclear factor kappa B
NLRP: nucleotide-binding oligomerization domain, leucine rich repeat and pyrin domain containing
OVA: ovalbumin
p62: sequestosome 1
PE: phosphatidylethanolamine
PI3K: phosphatidylinositol 3-kinase
PINK: PTEN-induced putative kinase
PTEN: phosphatase and tensin homologue
ROS: reactive oxygen species
SNAP: synaptosomal-associated protein
SNARE: soluble N-ethylmaleimide sensitive factor attachment protein receptor
SRT: sirtuin
T2: type 2 immunity
TBK: TANK binding kinase
TFEB: transcription factor EB
TGF: transforming growth factor
Th: T helper CD4+ lymphocyte
TLR: Toll-like receptor
TRM: tripartite motif protein
ULK: unc-51 like activating kinase
VPS: vacuolar protein sorting
